# Pregnancy and birth complications associations with long-term adverse maternal mental health outcomes: a systematic review and meta-analysis protocol

**DOI:** 10.12688/hrbopenres.13660.2

**Published:** 2023-04-24

**Authors:** Elizabeth O Bodunde, Daire Buckley, Eimear O'Neill, Gillian M. Maher, Karen Matvienko-Sikar, Karen O'Connor, Fergus P. McCarthy, Ali S. Khashan

**Affiliations:** 1 School of Public Health, University College Cork, Cork, Ireland; 2 INFANT Research Centre, University College Cork, Cork, Ireland; 3 Perinatal Mental Health, AMHS and CAMHS, University College Cork, Cork, Ireland; 4 RISE, Early Intervention in Psychosis Team, South Lee Mental Health Services, Cork, Ireland; 5 Department of Psychiatry and Neurobehavioral Science, University College Cork, Cork, Ireland; 6 Department of Obstetrics and Gynaecology, Cork University Maternity Hospital, Cork, Ireland

**Keywords:** Pregnancy, birth, complications, long-term, adverse mental health outcome

## Abstract

**Background:** Existing studies have established an association between pregnancy, birth complications, and mental health in the first few weeks postpartum. However, there is no clear understanding of whether pregnancy and birth complications increase the risk of adverse maternal mental outcomes in the longer term. Research on maternal adverse mental health outcomes following pregnancy and birth complications beyond 12 months postpartum is scarce, and findings are inconsistent.

**Objective:** This systematic review and meta-analysis will examine the available evidence on the association between pregnancy and birth complications and long-term adverse maternal mental health outcomes.

**Methods and analysis**: We will include cohort, cross-sectional, and case-control studies in which a diagnosis of pregnancy and/or birth complication (preeclampsia, pregnancy loss, caesarean section, preterm birth, perineal laceration, neonatal intensive care unit admission, major obstetric haemorrhage, and birth injury/trauma) was reported and maternal mental disorders (depression, anxiety disorders, bipolar disorders, psychosis, and schizophrenia) after 12 months postpartum were the outcomes. A systematic search of PubMed, Embase, CINAHL, PsycINFO, and Web of Science will be conducted following a detailed search strategy until August 2022. Three authors will independently review titles and abstracts of all eligible studies, extract data using pre-defined standardised data extraction and assess the quality of each study using the Newcastle-Ottawa Scale. We will use random-effects meta-analysis for each exposure and outcome variable to calculate overall pooled estimates using the generic inverse variance method. This systematic review will follow the Preferred Reporting Items for Systematic reviews and Meta-Analyses guidelines.

**Ethical consideration**: The proposed systematic review and meta-analysis is based on published data; ethics approval is not required. The results will be presented at scientific meetings and publish in a peer-reviewed journal.

**PROSPERO registration**: CRD42022359017

## Introduction

Pregnancy and childbirth complications are known to cause substantial morbidity and psychological distress in mothers ^1, 2^. Preterm birth, preeclampsia, preterm labour, prenatal haemorrhage, and gestational hypertension are among the significant pregnancy problems and results in maternal and neonatal morbidity and mortality ^3, 4^. According to the World Health Organization, in 15% of all pregnancies, a spectrum of pregnancy and birth complications may occur ^5^. Every year more than one and a half million women suffer from pregnancy-related complications during pregnancy and birth ^6^. Association between obstetric complications and chronic psychiatric and medical conditions in later life is becoming recognised ^7^. It has been established that these complications can lead to the emergence of stress and trauma, both of which can affect a woman’s mental state ^8^.

Several studies have explored short-term maternal mental health problems following pregnancy and birth complications with a focus on maternal postpartum stress ^9, 10^. Pregnancy and birth are potential triggers for new psychiatric illness, particularly after unexpected events, caesarean sections, miscarriages and, perception of negative or traumatic birth ^11^. Preterm birth is associated with an increased risk for depression, anxiety, and stress in the immediate postpartum period ^12– 14^. Other studies reported that spontaneous abortion and miscarriage during pregnancy puts women at higher risk for posttraumatic stress disorder (PTSD) and bipolar disorder at six to eight weeks postpartum ^15, 16^. Some studies suggested that having preeclampsia comorbidities resulted in the highest risk of psychiatric episodes ^17, 18^. A Nigerian study reported independent factors such as hospital admission, emergency caesarean section, and the poor maternal experience of control during childbirth to be associated with PTSD at six weeks postpartum ^19^. Several systematic reviews and meta-analyses have also suggested that adverse pregnancy and birth outcomes such as preeclampsia, preterm birth, and mode of delivery were risk factors for postpartum mental disorders ^20– 22^.

Neiger *et al.* 2017, reported that these obstetrical problems continue to impact on maternal health years after the index pregnancy ^23^. Postpartum stress and depression have frequently been investigated, and predictors that have been most associated are pre-existing psychiatric comorbidities, stress levels in pregnancy, and poor social support ^11^. Only a small number of studies have reported on maternal mental outcomes after pregnancy and birth complications, and most are restricted to the first few months of life ^24– 27^. Two studies with an average of seven years of follow-up showed inconsistent findings; when compared to a control group, Gaugler-Senden *et al.* reported PTSD symptoms in women who experienced early preeclampsia but no difference in depression and anxiety. Whereas Postma *et al.* found that women with early preeclampsia reported higher depressive and anxiety symptoms ^28, 29^.

Despite the psychological implications of adverse pregnancy and birth complications, the plausibility of associations with long-term adverse maternal mental health outcomes is yet to be systematically evaluated in existing literature, highlighting the need for further study in this area. Examining the association between pregnancy and birth complications and mental outcomes beyond the immediate postpartum period is an essential contribution to obstetric care and public health. The findings of this review will provide an overview of the current state of knowledge regarding whether having a complicated pregnancy or birth is a separate risk factor for adverse maternal mental health outcomes beyond the first year following childbirth. Therefore, this systematic review aims to synthesize the available evidence assessing the association between pregnancy and childbirth complications and long-term adverse maternal mental health outcomes.

## Review question

Do pregnancy and birth complications increase the risk of adverse maternal mental health outcomes after 12 months postpartum?

## Methods

The following PICO requirements will guide this systematic review.

### Population

Women who have had at least one pregnancy.

### Exposures

We will consider any of the following pregnancy and birth complication as exposures of interest: preeclampsia, pregnancy loss (miscarriage, stillbirth, and spontaneous abortion), caesarean section (elective, and emergency), preterm birth (defined as birth <37 weeks gestation), third/fourth perineal laceration, neonatal intensive care unit (NICU) admission >72 hours, major obstetric haemorrhage, birth injury/trauma. It should be noted that some of these complications such as elective caesarean section maybe at the request of the pregnant women and therefore may not be considered a complication. We plan to have a separate meta-analysis for each complication as we explained later.

### Comparison

Women who never had corresponding pregnancy and birth complications. For instance, we will compare women who experienced an emergency caesarean section to those who never experienced an emergency caesarean section.

### Outcomes

Any of the following adverse maternal mental health outcomes diagnosed or reported after the first year following childbirth will be considered an outcome of interest.

Primary outcomes: anxiety disorder, bipolar disorders, depression, schizophrenia, psychosis, and posttraumatic stress disorder.

Other outcomes: obsessive-compulsive disorder, panic disorder, agoraphobia, social phobia, acute stress disorder, delusional disorder, eating disorder, somatisation disorder, body dysmorphic disorder, conversion disorder, and substance-related disorder.

### Protocol and registration

This study adheres to the guidelines in the Preferred Reporting Items for Systematic Reviews and Meta-Analysis Protocols (PRISMA-P) statement ^30^. Under the guidelines, this systematic review protocol was registered with the International Prospective Register of Systematic Reviews (PROSPERO) on 11 September 2022 with registration number CRD42022359017.

### Search strategy

1.    One reviewer (E.O.B) will systematically search the literature in the following electronic databases: PubMed, CINAHL, EMBASE, PsycINFO and Web of Science, including all years from the inception of the electronic databases until August 2022. A detailed search strategy has been compiled and these terms will be searched according to the principles of Boolean Logic (AND, OR NOT) and using Medical Subject Headings (MeSH). The search strategy is included in the Extended data ^31^.

2.    The reference lists of the included studies will be manually searched to find additional potentially eligible research as a supplement to the electronic database searches.

### Criteria for considering studies for the review

***Inclusion criteria***

- Cohort, case-control, and cross-sectional studies in which a complication of pregnancy or childbirth was reported, and maternal mental health beyond the first year following childbirth is the outcome of interest.
- Data must be from an original study. If more than one study were based on the same dataset, the study with the longest follow-up period will be included. We may perform sensitivity analyses for different scenarios. Such analyses will be highlighted as post-hoc.
- A complication of pregnancy or childbirth and maternal mental health may be confirmed through medical records, doctor-diagnosed self-reporting, or validated questionnaires.
- We will include studies published in English only.
- Peer-reviewed literature only will be included.

***Exclusion criteria***

- Systematic reviews, case reports, case series, letters, commentaries, notes, editorials, conference abstracts and dissertations.
- Studies that are not published in English.
- Studies on women with a pre-existing mental illness or mental illnesses prior to 12 months postpartum.
- Animal studies.

### Selection of studies for inclusion in the review

Titles and abstracts of studies retrieved from each database search will be stored and managed in the EndNote reference manager and de-duplicated. Three review authors (E.O.B, E.O and D.B) will independently review the titles and abstracts of the studies. Full texts will be obtained where necessary to screen for eligibility in the systematic review and meta-analysis following the pre-defined inclusion/exclusion criteria. Where consensus on eligibility cannot be achieved, a fourth review author (A.S.K) will be involved in the discussion to reach a consensus. In the case of an eligible study, where more data is needed, the corresponding author will be contacted via email. If the corresponding author does not reply, a reminder will be sent two weeks later.

### Data extraction and management

Three reviewers (E.O.B, E.O, and D.B) will independently extract data from the eligible studies using a standardised data extraction form. We will extract data including the author and year of publication, study design, country and setting of study, sample size, definition or assessment of the exposures and outcome(s) of interest, comparison group, length of follow up, confounders adjusted for (if any), crude and adjusted estimates. Where necessary, we will contact corresponding authors of published studies to obtain relevant information about effect estimates. Discrepancies will be discussed between reviewers and where necessary, a fourth reviewer (A.S.K) will be consulted to achieve a consensus.

### Quality appraisal of included studies

Quality assessment of the included studies will be conducted by three reviewers (E.O.B, E.O, and D.B) independently and agreed upon subsequently using the Newcastle Ottawa Scale ^32^. This scale uses a “star system,” in which stars are assigned to show the quality of studies based on the following three criteria: selection of the study groups, comparability of the groups, and the ascertainment of the exposure and outcome of interest (the total score ranged from 0–9). We will consider 0 to 3 stars low quality, 4 to 6 stars moderate quality, and 7 to 9 stars high quality. The overall likelihood of bias will be assessed and reported for each study. Discrepancies will be discussed between reviewers and where necessary, a fourth reviewer (A.S.K) will be consulted.

### Data synthesis, including assessment of heterogeneity

We will undertake separate meta-analyses for each exposure-outcome association. Random effects meta-analyses will be performed to calculate overall pooled estimates where data allow. For example, for preeclampsia as an exposure of interest, a meta-analysis will be undertaken to investigate the association between (1) preeclampsia and anxiety disorders, (2) preeclampsia and bipolar disorders, (3) preeclampsia and schizophrenia. We will repeat this for all exposures. The generic inverse variance method will be used to display crude and adjusted results where possible. We will base the adjustment on the definition outlined in each identified study. We will also perform the following subgroup/sensitivity analyses where the data allow, using RevMan 5.4:

1) According to study design (cohort vs case-control vs cross-sectional).
2) According to the study quality (minimal/low versus moderate/high).
3) According to the measurement of outcome data (medical records versus doctor-diagnosed self-reported versus validated questionnaires).
4) According to the length of follow-up.

Publication bias will be assessed using a funnel plot, provided at least 10 or more studies are included in the meta-analysis. Where any other subgroup/sensitivity analyses are identified in the meta-analysis, such as analyses to explore potential high heterogeneity, these will be clearly labelled as post-hoc analyses.

### Presenting and reporting the results

A PRISMA flow diagram will be included to outline the step-by-step study selection process, and a rationale provided for excluded studies at full-text screening. The characteristics and quality assessment of the included studies will be presented in tables, and pooled estimates will be presented using forest plots. If raw data cannot be obtained for inclusion in meta-analyses, the findings will be included individually in a separate table. Where data is unsuitable for meta-analysis, study findings will be narratively synthesized and presented in tables.

## Conclusions

This systematic review and meta-analysis will summarise the existing literature examining the association between pregnancy and birth complications and long-term adverse maternal mental outcomes based on a prespecified study protocol. The high prevalence of pregnancy and birth complications suggests that any potential association would have important public health implications.

## Potential strengths and limitations of this study

The strength of this systematic review and meta-analysis includes providing updated knowledge on the associations between common pregnancy and birth complications and the risk of adverse maternal mental health outcomes in the long term. The use of a comprehensive search strategy, a prospectively registered protocol, and adherence to the PRISMA guidelines are further strengths of this review. In addition, three reviewers will screen for study eligibility and perform data extraction and quality appraisal of included studies, minimising the likelihood of reviewer-based bias in the systematic review.

We anticipate that publication bias may be a limitation in this review. Studies that show an effect have an increased likelihood of being published and published in English. Due to limited resources, the systematic review search will be confined to studies published in the English language only, potentially resulting in publication bias and relevant indexed studies being overlooked. Some studies may report more than one complication as an exposure, where exposure cannot be determined, we will narratively synthesize results and provide summary of results. If possible, a funnel plot will be used to assess the presence of publication bias. Furthermore, the presence of confounding is a major concern in observational studies. Potential confounders may include the family’s socio-economic status, maternal age, parity, body mass index (BMI), maternal smoking, and alcohol status during pregnancy. As mentioned above, our meta-analyses will display both crude and adjusted results where possible using the generic inverse variance method, basing adjustment on the definition outlined in each identified study.

## Ethics and dissemination

Given that this is a protocol for a systematic review based on published data, there is no requirement for ethics approval. It is anticipated that findings will be disseminated through publication in a peer-reviewed journal and conference presentations.

## Data Availability

No data are associated with this article. Figshare: Search strategy- Pregnancy and birth complications and long-term adverse maternal mental outcomes: a systematic review and meta-analysis protocol, https://doi.org/10.6084/m9.figshare.21572304 ^31^ Figshare: Preferred Reporting Items for Systematic review and Meta-analysis protocol (PRISMA-P) checklist and flow diagram. https://doi.org/10.6084/m9.figshare.21568716 ^30^ Data are available under the terms of the Creative Commons Attribution 4.0 International license (CC-BY 4.0).
