# Supplementary material for: Pregnancy and birth complications associations with long-term adverse maternal mental health outcomes: a systematic review and meta-analysis protocol
Source: HRB Open Res. 2023 Oct 24;6:3. Originally published 2023 Jan 10. [Version 3] doi: 10.12688/hrbopenres.13660.3 (PMC10636347; doi:10.12688/hrbopenres.13660.3)
Supplement: Peer review history

# Reviewer report: Reviewer response for version 3

**DOI:** 10.21956/hrbopenres.15135.r36759

**Published:** 2023-11-09

**Authors:** Christina Kang-Yi

**Affiliations:** 1 Rutgers Biomedical and Health Sciences, New Jersy, USA

The authors addressed all my comments, and I have no further comments to make.

Is the study design appropriate for the research question?

Partly

Is the rationale for, and objectives of, the study clearly described?

Yes

Are sufficient details of the methods provided to allow replication by others?

Partly

Are the datasets clearly presented in a useable and accessible format?

Not applicable

Reviewer Expertise:

Mental health services research

I confirm that I have read this submission and believe that I have an appropriate level of expertise to confirm that it is of an acceptable scientific standard.

# Reviewer report: Reviewer response for version 2

**DOI:** 10.21956/hrbopenres.15021.r35380

**Published:** 2023-09-07

**Authors:** Christina Kang-Yi

**Affiliations:** 1 Rutgers Biomedical and Health Sciences, New Jersy, USA

The study aims to synthesize the available evidence of association between pregnancy and childbirth complications and long-term adverse maternal mental health outcomes by systematically reviewing published observational studies including cohort, case-control, and cross-sectional studies. Given the limited information on maternal mental health outcomes resulted in by pregnancy and childbirth complications, the systematic review has potential to contribute to improving preventive maternal mental health.

 The major challenge in achieving the aim of the systematic review is identifying increased risk of mental health disorders 12 months after pregnancy and childbirth complications by reviewing observational studies that used medical records. Mental Health diagnoses identified through medical records do not reflect the exact onset of mental disorders for women with delayed help seeking and treatment. Thus, definitions of onset of mental health disorders identified through medical records will be arbitrary and can lead to inaccurate detection of mental health disorders 12 months after pregnancy and childbirth complications. Additionally, mental health diagnoses identified through administrative data such as health claims usually include a lag time. Lastly, studies based on using medical records will include a limitation of not knowing pre-existing mental health disorders, unless the studies retrospectively observe multiple years of data on mental health diagnoses prior to pregnancy. Please elaborate how you will handle the difficulties of identifying the increased risk of mental health disorders 12 months after pregnancy and childbirth complications, given these limitations of observational studies using medical records.

 There are other factors (other than the confounders authors listed in the protocol) that are associated with postpartum mental health disorders. For example, maternal self-efficacy, support by the partner, breastfeeding, a negative experience of the first week postpartum, and poor current health of the mother have been identified as significant factors associated with developing postpartum depression and/or anxiety. Refer to the following reference:

 van der Zee-van den Berg, A. I., Boere-Boonekamp, M. M., Groothuis-Oudshoorn, C. G. M., & Reijneveld, S. A. (2021). Postpartum depression and anxiety: a community-based study on risk factors before, during and after pregnancy. *Journal of Affective Disorders*, *286*, 158-165. https://doi.org/10.1016/j.jad.2021.02.062

 If there is not a sufficient number of studies published on each pregnancy and childbirth complication outcome, conducting meta-analysis for each outcome, adjusting for the confounding factors identified in the literature, is not feasible. Please elaborate how you will handle this.

Is the study design appropriate for the research question?

Partly

Is the rationale for, and objectives of, the study clearly described?

Yes

Are sufficient details of the methods provided to allow replication by others?

Partly

Are the datasets clearly presented in a useable and accessible format?

Not applicable

Reviewer Expertise:

Mental health services research

I confirm that I have read this submission and believe that I have an appropriate level of expertise to confirm that it is of an acceptable scientific standard, however I have significant reservations, as outlined above.

## References

[1] : Postpartum depression and anxiety: a community-based study on risk factors before, during and after pregnancy. *J Affect Disord* .2021;286: 10.1016/j.jad.2021.02.062 158-165 10.1016/j.jad.2021.02.062 33725615

# Reviewer report: Reviewer response for version 2

**DOI:** 10.21956/hrbopenres.15021.r33663

**Published:** 2023-05-03

**Authors:** David Ellwood

**Affiliations:** 1 School of Medicine and Dentistry, Gold Coast University Hospital, Griffith University, Gold Coast, Qld, Australia; 2 Maternal-fetal Medicine, Gold Coast University Hospital, Gold Coast, Qld, Australia

I am happy with the responses of the authors to my previous comments, and the resulting revisions to the manuscript and I have no further comments or questions.

Is the study design appropriate for the research question?

Yes

Is the rationale for, and objectives of, the study clearly described?

Yes

Are sufficient details of the methods provided to allow replication by others?

Partly

Are the datasets clearly presented in a useable and accessible format?

Not applicable

Reviewer Expertise:

Clinical academic practice in obstetrics/maternal-fetal medicine, with extensive research interests in severe complications of pregnancy (especially perinatal death), and post-partum recovery including physical and emotional outcomes.

I confirm that I have read this submission and believe that I have an appropriate level of expertise to confirm that it is of an acceptable scientific standard.

# Reviewer report: Reviewer response for version 1

**DOI:** 10.21956/hrbopenres.14938.r33322

**Published:** 2023-01-31

**Authors:** David Ellwood

**Affiliations:** 1 School of Medicine and Dentistry, Gold Coast University Hospital, Griffith University, Gold Coast, Qld, Australia; 2 Maternal-fetal Medicine, Gold Coast University Hospital, Gold Coast, Qld, Australia

This article is a proposal for a systematic review of the published scientific literature on pregnancy and birth complications and their association with long-term mental health outcomes. The stated aim is to look at each specified pregnancy complication, and each mental health outcome, and examine what support there is from the published literature for any association. The protocol is generally well-written, and uses an appropriate methodology. The research question is important, and I support their comments that this is an area that is under-researched and not well understood. I have four questions/comments that may help the authors in making some minor revisions to their protocol.

1. It is always interesting to discuss what is a pregnancy and birth complication, and whether some of the very common sequela are really complications or should be considered to be within the sphere of 'normal' outcomes. The list given includes some devastating outcomes such as stillbirth, but also includes elective caesarean section (which for some women may not be considered a complication but the only rational option). Perineal tears can vary from minor to very severe with life-long consequences. Have you considered refining the list of complications to only include severe, unexpected maternal and/or perinatal outcomes of pregnancy and birth?
2. Many women in the reported studies will have experienced more than one complication. For example, someone with pre-eclampsia, who has an emergency caesarean section, and whose baby is admitted to the NICU for 2 weeks. This begs the question as to which complication was causative, or is it the combination that counts? How will you be able to deal with this issue in your meta-analysis?
3. The list of mental health conditions includes a number which are very common and for many women a diagnosis will predate the first pregnancy. Are you only interested in women with a new diagnosis and no pre-existing mental health conditions?
4. A few minor points are that data is a plural noun, and birth is a better description than the term 'delivery'. Also, I found the opening paragraph a bit contradictory. If only 5-15% of women experience a complication, but some of those in the list to be studied are much more common than that, it sounds like the study population is potentially a very large proportion of the birthing population.

Is the study design appropriate for the research question?

Yes

Is the rationale for, and objectives of, the study clearly described?

Yes

Are sufficient details of the methods provided to allow replication by others?

Partly

Are the datasets clearly presented in a useable and accessible format?

Not applicable

Reviewer Expertise:

Clinical academic practice in obstetrics/maternal-fetal medicine, with extensive research interests in severe complications of pregnancy (especially perinatal death), and post-partum recovery including physical and emotional outcomes.

I confirm that I have read this submission and believe that I have an appropriate level of expertise to confirm that it is of an acceptable scientific standard, however I have significant reservations, as outlined above.
